# MAKING THE CASE FOR AGE INCLUSIVITY IN HIGHER EDUCATION

**DOI:** 10.1093/geroni/igac059.1062

**Published:** 2022-12-20

**Authors:** Lauren Bowen, Joann Montepare, Nina Silverstein, Susan Whitbourne, Celeste Beaulieu

**Affiliations:** University of Massachusetts Boston, Boston, Massachusetts, United States; Lasell University, Newton, Massachusetts, United States; University of Massachusetts Boston, Boston, Massachusetts, United States; University of Massachusetts Amherst, Amherst, Massachusetts, United States; University of Massachusetts Boston, Boston, Massachusetts, United States

## Abstract

Often viewed as institutions primarily servings 18-to-24-year-old student populations, U.S. colleges and universities are age-diverse. In our recent national study of AFU institutions, 21 campuses maintaining age data reported that 12,718 faculty (39.24% of faculty) and 20,361 staff (42.31% of staff) were ages 50+. Additionally, 22 campuses reported 7,080 students (1.58%) ages 50+. Despite higher education’s attention to diversity, equity, and inclusion (DEI), age is often overlooked; therefore, universities may need strategies for improving age inclusivity. Across 2,447 open-ended survey responses from our study, faculty, staff, and students describe experiences with age inclusivity (e.g., feeling valued) and exclusion (e.g., feeling unwelcome), and many call for greater sensitivity to aging in existing DEI efforts, such as more age-inclusive language in classrooms and attention to age bias in hiring and promotions. In addition, this presentation will examine responses that raise important considerations for integrating age inclusivity with other higher education DEI efforts.

